# The role of radiotherapy in small cell lung cancer: a new paradigm for the radiation oncologist

**DOI:** 10.3389/fonc.2024.1541527

**Published:** 2025-01-24

**Authors:** Paolo Borghetti, Sara Ramella, Umberto Ricardi

**Affiliations:** ^1^ Radiation Oncology Department, Azienda Socio Sanitaria Territoriale Spedali Civili and University of Brescia, Brescia, Italy; ^2^ Research Unit of Radiation Oncology Unit, Department of Medicine and Surgery, Università Campus Bio-Medico, Rome, Italy; ^3^ Radiation Oncology, Fondazione Policlinico Universitario Campus Bio-Medico, Rome, Italy; ^4^ Department of Oncology, University of Turin, Turin, Italy

**Keywords:** small cell lung cancer, radiotherapy, immunotherapy, multidisciplinary team, radiation oncologist

## Abstract

Small cell lung cancer (SCLC) is an aggressive tumor that presents in most cases as a metastatic disease. The prognosis is poor, but the advent of immunotherapy has rekindled hopes for outcomes. Radiotherapy plays a crucial role in this oncological scenario, and there are still many open questions on the correct application of radiotherapy and its integration with chemotherapy and immunotherapy. These issues are of great interest to the oncology community; among these, in particular, there are the choice of optimal fractionation and total dose for thoracic radiotherapy in limited SCLC and its biological implications, the role of prophylactic cranial irradiation and thoracic consolidation in the context of modern treatments with chemoimmunotherapy in extensive SCLC, the role and indications of stereotactic radiotherapy in oligometastatic scenario and finally the complex clinical and multidisciplinary management of SCLC. This perspective article aims to describe the strengths and limitations of the role of radiotherapy in SCLC, highlighting the critical role of radiotherapy and the radiation oncologist, with the need to implement specific knowledge and skills on SCLC.

## Introduction

Small cell lung cancer (SCLC) accounts approximately 12-15% of all of lung cancer cases (1). It is characterized by aggressiveness behaviour and high pronounced ability to develop distant metastases, resulting in a poor overall prognosis (2). However, recent advancement, after the introduction of immunotherapy, has improved the overall survival (OS) in both extensive-stage (ES-SCLC) and limited-stage (LS-SCLC) as shown in Caspian, IMpower133 and Adriatic trials (3–5). Historically, SCLC has been underrepresented in research and clinical innovation, leading to limited progress in improving local control and survival rates. Firstly, the lack of prospective registries has contributed to the scarcity of robust epidemiological data. However, the Surveillance, Epidemiology, and End Results (SEER) database provides valuable insights, showing an increase in incidence from 1975 until the mid-1990s, followed by stabilization until 2010, and a slight decline thereafter (6).

The incidence in females has progressively increased steadily and the average age of patients has increased from 64 years in the 1970s to 69 in the five-year period 2015-19/in recent years (6). These trends are closely tied to reduction in smoking habits in the United States since the 1990s, confirming the strong correlation between smoking and SCLC development (6, 7). Unfortunately, lung cancer screening programs have limited success in early SCLC detection. The sensibility of low dose computed tomography for early-stage SCLC remains significantly lower than for other lung cancer subtypes due to the aggressive biology and rapid growth of SCLC. Therefore, advanced-stage disease predominates and the role of surgery or stereotactic radiotherapy in stage I is still very limited (7). The availability of advanced staging tools, such as Positron Emission Tomography (PET) and brain Magnetic Resonance Imaging (MRI), has improved the accuracy of disease classification, contributing to an increase in the diagnosis of ES-SCLC. The introduction of the tumour-node-metastasis (TNM) staging system by the International Association for the Study of Lung Cancer (IASLC) provides a more significant approach compared to the dichotomous Veterans Administration Lung Cancer Study Group (VALSG) system. However, VALSG staging remains prevalent in clinical practice, and efforts should continue to transition to the TNM system for a more precise assessment.

Despite these advances, the prognosis of patients with SCLC has remained largely unchanged over the past four decades, with median survival rates of 6-8 months. Male patients and those aged over 70 years represent disadvantaged subgroups (6). It should be noted that these data cannot be representative of the current prognosis of patients with SCLC, as they do not adequately reflect the advances in treatments introduced since 2020.

SCLC remains a complex disease to manage due to the intrinsic fragility of patients who are often elderly and present significant comorbidities (8). Immunotherapy has reinvigorated clinical and scientific interest in SCLC, highlighting the enduring strategic importance of radiotherapy in both ES-SCLC and LS-SCLC. The radiotherapy community continues to question itself on the various issues related to the radiotherapy prescription in integrated treatment with chemotherapy in LS-SCLC, in thoracic consolidation role in ES-SCLC, in prophylactic cranial irradiation (PCI) and in the treatment of metastases with particular reference to oligometastatic setting. The aim of this prospective article is to present the most up-to-date data on these unresolved issues in radiotherapy, to provide concrete elements for engaging multidisciplinary discussion. The clinical complexity combined with the emerging therapeutic innovations, necessitates a tailored and collaborative approach. Radiotherapy, integrated within a multidisciplinary framework, remains crucial for enhancing treatment efficacy while minimizing adverse effects.

## Materials and methods

This literature review focused on a critical and non-systematic analysis of scientific works published exclusively in recent years in order to provide detailed and updated information on the different issues addressed.

## Results

### The debate on dose fractionation: a question of biology

In 2024, Du et al. published an extensive review of the literature focusing on the impact of dose fractionation and total dose on OS in patients treated with radiochemotherapy for LS-SCLC disease (9). The authors correlated the two-year survival rate with the biologically effective dose (BED) using the equation that correlates the BED with the dose per fraction, the α/β coefficient (set to 10), the treatment time and the disease doubling time (10). They showed a large number of experiences that have adopted different dose fractionations over the years: conventional, hypofractionated, hyperfractionated or stereotactic. They essentially demonstrated how the 2-year OS with the use of conventional fractionation schedules has remained almost stable in the different published series (9). The studies that adopted hyperfractionated schedules observed an increasing rate of OS at 2 years when associated with dose escalation. Interestingly, hypofractionated dose schedules also showed very promising results. **Table 1** summarizes the most relevant scientific trials and compares the 2-year OS with the BED10 delivered. The Turrisi schedule, still considered the current standard, allowed to obtain median OS at 2 years of 47% using the dose of 45 Gy delivered in 30 fractions of 1.5 Gy twice daily (11). The Convert trial did not demonstrate a non-inferiority of this “Turrisi” schedule, even when compared to a dose of 66 Gy in 33 fractions (12). Not even the RTOG 0538 study, based on the rationale of dose escalation, showed significant advantages in 2-year OS by delivering 70 Gy in 35 fractions rather than the consolidated Turrisi schedule although the BED10 was higher (13).

**Table 1:** Phase 2 and 3 trials comparing 2-year OS with the BED10 across different fractionation schedules.

Trial	Phase	RT schedule Gy/fr	Fraction per day	BED10 (Gy)	2-year OS (%)
AT Turrisi (Intergroup 0096) 1999 (11)	3	45/25 45/30	1 2	39,49 43,91	41 47
C Faivre-Finn (Convert) 2017 (12)	3	45/30 66/33	2 1	43,91 60,64	56 51
J Bogart (CALGB 30610(Alliance)/RTOG 0538) 2023 (13)	3	45/30 70/35	2 1	43,91 64,61	58 56
BH Grønberg 2016 (14)	2	45/30 42/15	2 1	43,91 45,92	53 42
B Qiu 2021 (10)	2	45/30 65/26	2 1	43,91 66,40	70 74
BH Grønberg 2021 (15)	2	45/30 60/40	2 2	43,91 58,28	48 74
J Yu 2024 (16)	3	45/30 54/30	2 2	43,91 55,88	54 76

RT, radiotherapy; BED, Biologically Effective Dose; OS, Overall Survival.

The possibility of glimpsing an improvement in terms of OS occurred with the publication of two phase 2 studies in 2021 (10, 14). They introduced accelerated hyperfractionation in 40 fractions and hypofractionation in 26 fractions achieving median OS of 74% at 2 years by delivering BED10 of 58.28 Gy and 64.40 Gy, respectively (10, 14, 15). Recently, a phase 3 randomized trial showed a significant better OS when 54 Gy in 30 fractions twice daily versus 45 Gy in 30 fractions twice daily were delivered as simultaneous integrated boost on gross target volume (16).

In conclusion, it could be argued that the conventional dose fractionation regimen even in dose escalation studies is not the best strategy to pursue since it is negatively affected by the lengthening of the total treatment time and is strongly influenced by rapid cellular repopulation. On the contrary, in the future, the options of hypofractionation or hyperfractionation associated with dose escalation should be further explored in phase 3 studies with an integrated approach in the light of the recent reported benefit of immunotherapy after chemoradiation. Hypofractionation could probably be preferred because it would be more convenient both for logistical reasons (less access to hospital facilities and reduced occupation of the linear accelerator) and for compliance reasons (better quality of life, lower costs). However, it should be noted that concurrent hypofractionated radiotherapy and chemotherapy could be burdened by higher toxicity rates. Therefore, at the moment, the hyperfractionated strategy should still play a central role.

### Thoracic consolidation: a matter of choice

Thoracic radiotherapy (TRT) as “consolidation” treatment in extensive stage was investigated in the CREST study published by Slotman et al. in 2015 (17). This study, while not reaching the primary endpoint established in one-year survival, had nevertheless demonstrated a significant benefit in 2-year OS. A subsequent 2016 meta-analysis combined the study by Slotman et al. that used 30 Gy TRT in 10 fractions with that of Jeremic et al. from the late 1990s that used a twice-daily dose of 54 Gy. This meta-analysis confirmed a significant benefit in the use of TRT after chemotherapy in extensive small cell lung cancer (18, 19). A further meta-analysis that also considered retrospective studies and the retrospective collection of the USA National Cancer Database allowed the same results to be reiterated (20, 21). The real current limitation of the evidence presented so far lies in the fact that none of these works included the current standard of primary treatment of ES-SCLC, which involves now the combination of chemo-immunotherapy. For this reason, the adoption of TRT in patients with response after systemic treatment needs to be reevaluated in light of the change in medical therapy strategy which includes atezolizumab or durvalumab in as maintenance therapy after chemotherapy with platinum and etoposide. In 2024 Feng et al. published the results of a systematic review that included a total of 1033 patients from 15 studies (3 prospectives, 9 with dual-arm cohorts) where the impact of TRT in patients treated with chemoimmunotherapy was still observed. Even in this updated setting, the option of TRT on the thoracic remnant after chemoimmunotherapy apparently remains valid with a HR of 0.52 (0.39-0.68). This benefit translates into better OS and progression-free survival (PFS) at six months and one year without significant impact in terms of toxicity, especially for radiation pneumonitis (22), with the use of “palliative” schedules (normally 30 Gy in 10 fractions) or with higher total doses.

Based on the data presented above, TRT could actually be able to improve clinical outcomes for patients with ES-SCLC. In particular, it could play a crucial role in increasing local control, reducing the probability of thoracic progression, which is often responsible for severe symptoms. However, some uncertainties remain regarding the correct selection of patients, the initial disease burden, the optimal dose to be administered and the timing with respect to systemic therapy. It is possible to believe that patients who benefit most from TRT are those with a better performance status, who obtain a better response to chemotherapy or chemo-immunotherapy and present a limited disease burden. For these patients, a more aggressive treatment proposal may be offered, including PCI, as reported in the NRG study (23). On the other hand, it may be superfluous to propose thoracic consolidation in patients with a complete response to systemic treatment. In the era of immunotherapy, the use of radiotherapy treatment concomitant with immune checkpoint inhibitors must be evaluated with caution when large lymph node volumes are included in the target. For this reason, it would be advisable to propose TRT at doses not exceeding 30 Gy in 10 fractions and limiting only to residual disease to be treated with high dose gradient techniques.

### PCI: a question of method

PCI remains a widely debated issue in the context of SCLC treatment choices, particularly because of the emerging role of brain MRI, neurotoxicity, the advent of immunotherapy and the increasing use of stereotactic brain radiotherapy. In 1999, Auperin et al. showed for the first time the benefit in both survival and incidence of brain metastases obtained by PCI in patients with limited and extensive disease after complete response to chemotherapy (24). Slotman et al. in 2007 confirmed the same data also for extensive disease after any response to chemotherapy (25). In these trials brain imaging was not always part of standard staging and follow-up procedures, unless symptoms suggestive of brain metastases were present. Takahashi et al. in 2017 introduced the concept of radiological follow-up with MRI as an alternative to PCI, demonstrating that in ES-SCLC there was no significant difference in terms of survival between PCI and brain MRI at 3-month intervals up to 12 months and at 18 and 24 months after enrolment (26). Between 2023 and 2024, three different meta-analyses on this topic were published, confirming its scientific and clinical relevance (27–29). The first meta-analysis of 2023 includes 74 studies for a total of 31,551 patients, of which 26.7% were treated with PCI. This meta-analysis concluded supporting the benefit of PCI in terms of OS, PFS, brain metastasis-free survival (BMFS) in both LS-SCLC and ES-SCLC. However, this benefit is not confirmed when brain MRI is applied before PCI in cases of diseases-SCLC (27). The second meta-analysis, involving patients with LS-SCLC only, included 10 retrospective studies for a total of 532 patients treated with PCI and 613 not receiving PCI. Cheng et al. concluded that PCI improved OS and PFS and reduced the risk of brain metastases independently of the use of MRI (28). Finally, the third meta-analysis on 56,770 patients from 223 studies reaffirmed the positive impact of PCI except when brain MRI was used in both LS-SCLC and ES-SCLC (29). In summary, in the clinical practice, MRI should always be performed before making any decision regarding the opportunity to propose PCI, knowing that this could increase the costs, complexity and duration of the staging phase.

Unfortunately, neurological toxicity in terms of cognitive decline has a negative impact on the outcome of these treatments and 3randomized studies have attempted to investigate the role of PCI with avoidance of the hippocampus (HA-PCI) as a more refined method capable of limiting neurological toxicity (30–32). Belderbos et al. failed to achieve the protective effect by not demonstrating any beneficial effect on cognitive function after HA-PCI (30). Similarly, Albers et al. did not demonstrate any advantage in terms of preservation of cognitive function and quality of life after HA-PCI (31). The only opposite data against the trend is that of De Dios et al. who seems to show a preservation of cognitive function without impacting the clinical outcomes of intracranial failure and OS (30). At ASTRO 2023 a phase IIR/III study was presented demonstrating a relative risk reduction of 23% of first failure in any cognitive tests, even if no benefit for the primary endpoint has been reported (33). Recently, a pooled longitudinal individual patient data of two phase III trials, NCT01780675 and PREMER, has been published in order to investigate whether HA-PCI is associated with improved self-reported cognitive functioning compared with PCI without increasing brain metastases development within the HA area. The authors concluded that HA-PCI did not preserve longitudinal self-reported cognitive functioning but did also not increase the risk of BM (34). To date, HA-PCI cannot be considered a standard practice since the protective benefit in terms of neurocognitive decline is doubtful. However, since there is no increase in the risk of intracranial relapses and pragmatically it could be considered a valid alternative to traditional PCI, although aware that it is a procedure that requires time and greater technological effort.

Again, as with TRT, precise information is not available regarding the role of PCI in chemoimmunotherapy era. In particular, in extended disease, the two pivotal studies that led to the use of atezolizumab and durvalumab in this setting presented some intrinsic differences. Among the inclusion criteria of the IMpower133 study there was the presence of asymptomatic and treated brain metastases, while in the Caspian study brain metastases could be asymptomatic or treated (3, 4, 35, 36). In any case, the percentage of patients with brain metastases in the two studies was very limited, in a range between 8.5 and 14.8%. PCI was allowed in both arms in the IMpower study and only in the control arm in the Caspian study, but it was used in approximately 8-10% of cases. Finally, only the IMpower 133 study included a stratification for brain metastases. It is therefore not possible to clearly deduce from these studies the impact of PCI on survival (35, 36) (**Table 2**). Hopefully, a definitive answer to this question will be provided by the two ongoing phase 3 studies: MAVERIK (NCT04155034) and PRIMAlung Study (NCT04790253). Both studies compare the use of PCI with brain MRI surveillance in both LS-SCLC and ES-SCLC. The primary endpoint is OS and the secondary endpoint is the impact on neurocognitive decline. Interestingly, the first study includes the possibility of adopting HA techniques and both studies include immunotherapy as systemic therapy. In this perspective, it would be appropriate to evaluate on a case-by-case basis whether the use of PCI is appropriate, considering the patient’s age, performance status, response and tolerance to treatment and the presence of comorbidities that may favour the onset of cognitive decline (heavy smoking history, cerebrovascular disease). Last but not least, the benefits and disadvantages that could derive from the treatment must be clearly shared with the patient and caregivers.

**Table 2:** Impact of BM and use of PCI in 2 chemoimmunotherapy trials in extensive SCLC.

	Impower 133	Caspian
ICI	Atezolizumab	Durvalumab
Brain metastases (inclusion criteria)	Asymptomatic and treated	Asymptomatic or treated
Presence of brain metastases	8,5% Exp arm 8,9% Placebo arm	10,7% Experimental arm 14.8% Placebo arm
PCI	Both arms permitted	Only in control arm permitted
Frequency of PCI	10%	8%
Stratification for BM	yes	no
mOS (with BM)	8,5 mOS Experimental arm 9,7 mOS Placebo	11,7 mOS Experimental arm 8,8 mOS Placebo

ICI, Immune Checkpoint Inhibitor; BM, Brain Metastasis; mOS, median Overal Survival; PCI, Prophylactic Cranial Irradiation.

In general, it is observed that radiation oncologists are increasingly aware that intracranial progression can be managed with a local ablative treatment such as stereotactic radiotherapy (37). The possibility of following patients over time with MRI and the almost ubiquitous availability of performing stereotactic treatments has led to a reduction in the space of prophylactic treatment in favour of local treatment on metastases. This scenario has taken hold since the concept of oligometastasis can also be applied to SCLC as will be described in the next session.

### Oligometastasis: a question of logic

The treatment of oligometastases through the use of local ablative therapies has become of great interest and widespread use in common clinical practice for many oncological diseases (38). SCLC has long remained orphaned of data with respect to this treatment opportunity due to the poor prognosis and the idea that the high probability of distant progression did not make it susceptible to local treatment, except for palliative purposes only. Shirakawa et al. in 2019 showed for the first time that patients with a solitary metastasis in a single organ or with 2-5 metastases in a single organ had a better OS than patients with ≥ six metastases in a single organ or two or more organs involved (39). The RTOG 0937 trial designed to investigate the role of PCI plus or minus TRT in patients with 1-4 metastases and without encephalic involvement was closed early due to futility at an interim analysis. This meant that there was no adequate follow-up to validate the study endpoint, however the survival results of patients with extensive disease limited to a maximum of four metastases were substantially comparable to those of limited disease (23). Once again, the stereotype that SCLC with a low metastasis burden is comparable in terms of survival to the entire totality of ES-SCLC is broken. A significant contribution to the use of stereotactic ablative treatment (SRS) in SCLC with brain metastases was provided by Rusthoven et al. with the presentation of data from the Fire Cohort (40). In this study, patients with brain metastases treated with SRS and whole-brain radiotherapy (WBRT) were compared using a propensity score matching statistical method. The results of this study showed a statistically significant superiority in terms of OS with the use of stereotactic radiotherapy despite an increased cumulative incidence for time to central nervous system progression (TTCP) (40). A meta-analysis on the same topic echoed Rustoven’s study confirming the favourable impact in terms of OS when stereotactic radiotherapy is adopted as a treatment choice for brain metastases (41). As regards stereotactic ablative radiotherapy for non-brain lesions (SBRT), in 2023 the first series of 20 patients treated on 24 non-brain lesions (mainly lung metastases) in patients with oligoprogressive or oligorecurrent disease was published. The results appeared encouraging with no local failures despite a high rate of distant relapses (42), once again confirming efficacy and safety of ablative treatments, as well as the high systemic progression risk. A subsequent numerically more consistent experience was published in 2024 involving 93 patients with brain and extra-brain metastases in the context of SCLC with synchronous, metachronous or oligoprogressive oligometastases. The median survival was 14 months, with brain metastases or multiple metastases found to be factors independently related to a worse prognosis in the multivariate analysis (43). The advent of immunotherapy has also given an innovative push in this direction. If until recently, proposing a stereotactic treatment to a patient affected by SCLC was unthinkable, today it is at least an option to be considered with the aim of postponing a new systemic therapy or continuing with maintenance immunotherapy. It is possible to believe that the benefit obtained by stereotactic radiotherapy in the data presented above is linked to a better selection of patients such as long-survivors. Unfortunately, in the absence of prospective and randomized data, it is difficult to establish the real impact on PFS and OS, therefore the knowledge acquired in other oncological scenarios, where stereotactic radiotherapy is widely adopted on oligometastases, can be transferred with caution also to SCLC.

### SCLC management: a clinical issue

SCLC is a difficult and complex disease to manage and requires a multidisciplinary intervention starting from the diagnostic-staging framework to the therapeutic management and subsequent follow-up. The clinical presentation of patients with SCLC is commonly symptomatic and very often diagnostic suspicion occurs through access from the emergency room. In the earliest stages it is essential to be timely and effective in quickly reaching the diagnosis and this requires extensive involvement of all the actors involved in the diagnosis and staging of the disease (pulmonologist, radiologist, pathologist, general practitioner) (44). Currently, the role of the thoracic surgeon is considered marginal since the diagnosis of stage I SCLC is very rare. The definition of facilitated and personalized pathways is mandatory to avoid the patient experiencing a worsening of the general conditions that could then prevent access to chemotherapy, radiotherapy and immunotherapy treatments. Therefore, the multidisciplinary contribution of clinical oncologists (medical oncologists and radiation oncologists) is essential to establish the modalities and timing of the different therapeutic actions (45).

### New frontiers: a question of research

Today, patients with SCLC could receive more and more opportunities for treatment with radiotherapy as an expression of greater sensitivity and attention by the radiation oncologist to the different clinical scenarios in which SCLC occurs. For example, stereotactic radiotherapy could be proposed in early-stage disease when not amenable to surgery, as presented by a recent analysis. In this systematic review and meta-analysis, 1- and 2-year OS was 86.3% and 63.7% after stereotactic radiotherapy for stage I inoperable SCLC. The rates of chemotherapy and PCI were 44.1% and 13.8%, respectively. These findings shows that patients with inoperable early-stage, node-negative SCLC could be effectively approached with ablative radiotherapy combined with chemotherapy, if possible, and omitting PCI (46). Furthermore, the option of post-operative radiotherapy (PORT) could be evaluated for those few patients undergoing surgical resection as first therapy. In 2024, Zhang et al. presented a meta-analysis demonstrating a potential role of PORT in patients undergoing surgery and lymphadenectomy with pN2 mediastinal involvement (47).

The Adriatic study was very successful in demonstrating that maintenance durvalumab after chemoradiation in limited disease significantly improved patient survival (5). Durvalumab as maintenance led to significantly longer overall survival than placebo (median, 55.9 months [95% confidence interval {CI}, 37.3 to not reached] vs. 33.4 months [95% CI, 25.5 to 39.9]; hazard ratio for death, 0.73; 98.321% CI, 0.54 to 0.98; P = 0.01), as well as to significantly longer progression-free survival (median 16.6 months [95% CI, 10.2 to 28.2] vs. 9.2 months [95% CI, 7.4 to 12.9]; hazard ratio for progression or death, 0.76; 97.195% CI, 0.59 to 0.98; P = 0.02). A subgroup analysis presented at the 2024 ESMO Congress highlighted the positive contribution of twice-daily hyperfractionated radiotherapy and PCI to OS and PFS in both patients receiving durvalumab and placebo (48). On the contrary, the attempt to integrate immunotherapy with atezolizumab with concomitant radio-chemotherapy has been unsuccessful as shown in NRG Oncology/Alliance LU005 (49).

Many studies are currently underway to try to answer the many open questions with particular reference to the role of TRT and PCI in patients treated with modern standards of care, including the combination of chemotherapy and immunotherapy (ClinicalTrials.gov identifiers: NCT04155034, NCT04790253, NCT04829708, NCT04402788, NCT04462276). Another line of research that would be worth exploring is in the direction of a better understanding of the molecular mechanisms underlying this pathology. The absence of oncological drivers and biomarkers, unlike what happened in non-small cell lung cancer, still makes the biology of this pathology unknown and consequently the availability of truly effective drugs as scarce.

## Conclusion

Compared to the past, today SCLC enjoys greater attention and scientific interest thanks to the advent of immunotherapy that has revolutionized therapeutic strategies. The integration of systemic treatments with radiotherapy remains crucial. It is essential to increase the biological, technological and clinical skills of radiation oncologists to more effectively address the treatment path of patients with SCLC. The great innovations described as well as the persistent uncertainties make SCLC the paradigm of the pathology that sees the radiation oncologist at the center of many crucial decisions, responsible for complex therapeutic choices that require all possible efforts to fully understand the biology of this pathology, the best technological use to increase the benefit/risk ratio and clinical competence in knowing how to manage patients with SCLC.

## Data Availability

The original contributions presented in the study are included in the article/supplementary material. Further inquiries can be directed to the corresponding author.
